# Dexamethasone intravitreal implant in the crystalline lens: a case report

**DOI:** 10.5935/0004-2749.20200065

**Published:** 2020

**Authors:** Elif Ertan, Reşat Duman, Rahmi Duman, Mustafa Doğan

**Affiliations:** 1 Department of Ophthalmology, Siirt Kurtalan State Hospital, Siirt, Turkey; 2 Department of Ophthalmology, School of Medicine, Afyon University of Health Sciences, Afyonkarahisar, Turkey; 3 Department of Ophthalmology, Liv Hospital, Ankara, Turkey

**Keywords:** Macular edema, Diabetic retinopathy, Dexamethasone/administration & dosage, Intravitreal injections, Lens, crystalline, Drug implants, Visual acuity, Humans, Edema macular, Retinopatia diabética, Dexametasona/administração & dosagem, Injeções intravítreas, Cristalino, Implantes de medicamentos, Acuidade visual, Humanos

## Abstract

This report describes the therapeutic effects and outcomes of an accidental injection of an intralenticular sus tained-release dexamethasone implant (Ozurdex^®^) in three patients with diabetic macular edema. All three patients underwent accidental injections of sustained-release intravitreal dexamethasone implants into the crystalline lens by the same surgeon. After the accidental injection of Ozurdex^®^ into the crystalline lens, a remarkable reduction in the macular edema and an improvement in visual acuity were observed, suggesting that a positive outcome can be achieved without immediate surgery.

## Abstract (pt)

Este estudo descreve os efeitos terapêuticos e os resultados de uma injeção acidental de implante intralenticular de dexametasona de liberação sustentada (Ozurdex^®^) em três pacientes com edema macular diabético. Todos os três pacientes foram submetidos a injeções acidentais de implantes intravítreos de dexametasona de liberação sustentada na lente do cristalino pelo mesmo cirurgião. Após a injeção acidental de Ozurdex^®^ na lente do cristalino, observou-se uma redução notável no edema macular e uma melhora na acuidade visual, sugerindo que um resultado positivo pode ser alcançado sem cirurgia imediata.

## INTRODUCTION

Ozurdex^®^ (Allergan, Inc., Irvine, CA, USA) is a dexamethasone implant that is approved for the treatment of macular edema due to branch or central retinal vein occlusion, diabetic macular edema (DME), and noninfectious uveitis affecting the posterior segment^(1)^. Ozurdex^®^ is associated with ocular side effects, including conjunctival hemorrhage, endophthalmitis, cataracts, glaucoma, and retinal detachment^(2)^. Accidental injections of Ozurdex^®^ into the crystalline lens have rarely been described in the literature^(3,4)^. In this report, we describe the cases of three patients who experienced lens damage following the injection of a dexamethasone implant for the treatment of DME.

## CASE REPORT

### Case 1

**Figure 1:**
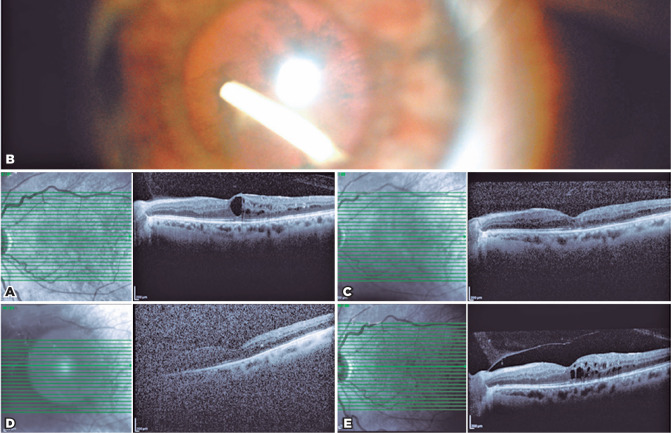
Case 1. (A) Macular OCT prior to Ozurdex®. (B) Intralenticular Ozurdex®. (C) Macular OCT at one month after accidental Ozurdex® injection. (D) Macular OCT prior to surgery. (E) Macular OCT at one month postoperatively.

A 56-year-old woman presented with DME. Her best-corrected visual acuity (BCVA) measured 20/200, intraocular pressure (IOP) was 13 mmHg, and central macular thickness (CMT) was 400 µm in the left eye (Figure 1A). She had undergone five previous intravitreal injections of ranibizumab for her DME. Ozurdex^®^ implant injection was performed owing to the presence of persistent DME. Following topical anesthesia with proparacaine, the implant was injected via the pars plana route 3.5 mm from the limbus. At one month after injection, slit-lamp examination revealed that the implant was located in the inferotemporal quadrant of the lens (Figure 1B) and that macular edema had resolved. Optical coherence tomography (OCT) scanning showed that the CMT had regressed from 400 to 266 µm (Figure 1C). Additionally, her BCVA measured 20/100 and IOP was 18 mmHg in the left eye. Conservative management was continued in order to determine whether the implant could be effective in the treatment of her macular edema. At three months after injection, the CMT increased from 266 to 320 µm (Figure 1D) and her BCVA decreased from 20/100 to 20/200. Surgery was planned because of cataract progression. Phacoemulsification surgery was performed at three months after injection. During phacoemulsification, Ozurdex^®^ was divided with the crystalline lens and aspirated with a phacoemulsification probe. During phacoemulsification, posterior capsulorhexis was performed and a monofocal hydrophobic 24.00 D intraocular lens (IOL) was placed in the bag. The IOL was well centered at one week postoperatively, and the IOP was within the normal limit. At one month postoperatively, her BCVA measured 20/125, IOP was 16 mmHg, and CMT was 341 µm in the left eye (Figure 1E).

### Case 2

**Figure 2:**
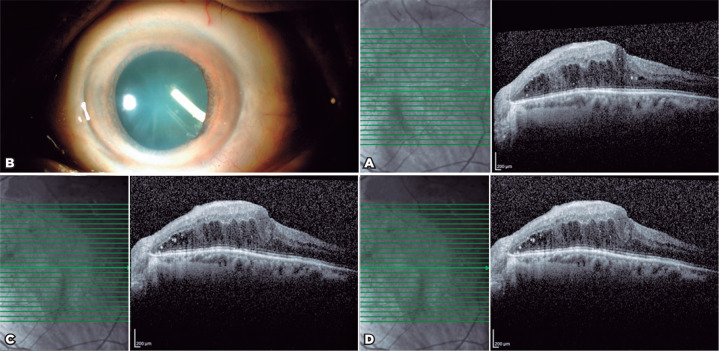
Case 2. (A) Macular OCT prior to Ozurdex®. (B) Intralenticular Ozurdex®. (C) Macular OCT at one month after accidental Ozurdex® injection. (D) Macular OCT at one month postoperatively.

A 78-year-old man presented with DME. His BCVA measured 20/100, IOP was 18 mmHg, and CMT was 506 µm in the left eye (Figure 2A). He had previously received 10 intravitreal injections of ranibizumab for his DME. Owing to the presence of recurrent DME, Ozurdex^®^ implant injection was performed. The implant was injected via the pars plana route 3.5 mm from the limbus, following topical anesthesia with proparacaine. At one month after injection, slit-lamp examination revealed that the implant was located in the inferotemporal quadrant of the lens (Figure 2B). Furthermore, his BCVA measured 20/125 and CMT did not decrease (Figura 2C). At the two-month follow-up, cataract progression requiring intervention was observed and his macular edema remained unresolved. His preoperative BCVA measured 20/160 and CMT was 454 µm. During phacoemulsification, Ozurdex^®^ was divided with the crystalline lens and aspirated with a phacoemulsification probe. A monofocal hydrophobic 19.00 D IOL was placed in the bag. One day postoperatively, the IOL was well centered and the IOP was within the normal limit. At one month postoperatively, his BCVA measured 20/160 and CMT was 460 µm (Figure 2D).

### Case 3

**Figure 3:**
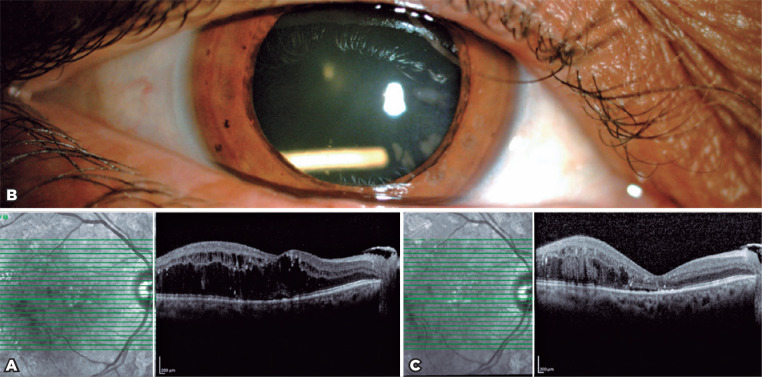
Case 3. (A) Macular OCT prior to Ozurdex®. (B) Intralenticular Ozurdex®. (C) Macular OCT at one month after accidental Ozurdex injection®.

A 57-year-old woman presented with DME (Figure 3A). Her BCVA measured 20/200 and IOP was 19 mmHg in the right eye. She had received 13 previous intravitreal injections of ranibizumab for her DME. Ozurdex^®^ implant injection was performed owing to the presence of recurrent DME. The implant was injected via the pars plana route 3.5 mm from the limbus, following topical anesthesia with proparacaine. At one month postoperatively, slit-lamp examination revealed that the implant was located in the inferotemporal quadrant of the lens (Figure 3B). Moreover, her macular edema had resolved and OCT scanning revealed that her CMT had decreased from 560 to 237 µm (Figure 3C), with the IOP being within the normal limit. Conservative management was continued to determine the effectiveness of the implant. At five months after injection, her CMT had increased from 237 to 353 µm, BCVA measured 20/400, and IOP was 20 mmHg. There was significant cataract progression; therefore, phacoemulsification surgery was performed at five months post-after injection. During phacoemulsification, posterior capsulorhexis and anterior vitrectomy were performed and a three-piece hydrophobic 21.00 D IOL (AR40E, AMO; Abbott) was placed in the sulcus. The IOL was well centered at one day postoperatively, and her IOP was within the normal limit. At one month postoperatively, her BCVA measured 20/200 and CMT was 353 µm.

## DISCUSSION

Accidental injection of a sustained-release intravitreal dexamethasone implant into the crystalline lens is an uncommon complication that may be due to inexperience, using an improper technique, and/or uncontrolled head movement during injection. There have been a few case reports regarding this complication^(5,6)^. Fasce et al. described two patients who experienced lens damage following the injection of dexamethasone implants for the treatment of macular edema associated with central retinal vein occlusion. At one week after injection, cataract extraction was performed in the first patient^(7)^. Phacoemulsification surgery was performed at two months after injection in the second patient.

In our second patient, the macular edema was unresolved, presumably because the dexamethasone implant had not protruded as far into the vitreous cavity as it had in the first and third patients. Abdolrahimzadeh et al. reported the intralenticular retention of a dexamethasone implant in a patient who demonstrated resolution of macular edema and lack of cataract progression within the eight-month follow-up period^(8)^. Se keroglu et al. reported the accidental injection of a sustained-release intravitreal dexamethasone implant into the crystalline lens in one patient; notably, the patient underwent phacoemulsification surgery at seven months after injection^(5)^. These reports provide evidence of the therapeutic efficacy of inadvertent intralenticular injection of Ozurdex^®^. In our first and third cases, the macular edema had resolved, presumably because the dexamethasone implant was only partially inside the lens and protruded into the vitreous cavity; therefore, the implants continued to be effective in the eye. In the first patient, phacoemulsification surgery was performed at three months after injection because of cataract progression. In the third patient, phacoemulsification surgery was performed at five months after injection, also because of cataract progression.

A frequent treatment using intralenticular Ozurdex^®^ is to perform immediate phacoemulsification with IOL implantation and then reposition the Ozurdex^®^ into the vitreous cavity^(4)^. An alternative approach is conservative management, in which phacoemulsification is not immediately performed if there is no significant decrease in the macular edema and an improvement in visual acuity is observed.

Here, we have described three patients who experienced an accidental injection of sustained-release intravitreal dexamethasone implants into the crystalline lens by the same surgeon; this complication was a result of the surgeon’s inexperience. Importantly, this type of complication can be prevented by thorough technical training on a reassured patient. After an accidental injection of Ozurdex^®^ into the crystalline lens, a remarkable reduction in macular edema and an improvement in visual acuity may also be achieved without immediate surgery.
